# Metastasis-associated in colon cancer-1 and aldehyde dehydrogenase 1 are metastatic and prognostic biomarker for non-small cell lung cancer

**DOI:** 10.1186/s12885-016-2903-z

**Published:** 2016-11-10

**Authors:** Lei Zhou, Lan Yu, Bo Zhu, Shiwu Wu, Wenqing Song, Xiaomeng Gong, Danna Wang

**Affiliations:** Department of Pathology the First Affiliated Hospital of Bengbu Medical College, Bengbu Medical College, No.287, Changhuai Road, Anhui Province, Bengbu, 233003 China

**Keywords:** NSCLC, MACC1, ALDH1, CSCs, Prognosis

## Abstract

**Background:**

Tumor recurrence and metastasis are the most common reason for treatment failure. Metastasis-associate in colon cancer-1 (MACC1) has been identified as a metastatic and prognostic biomarker for colorectal cancer and other solid tumors. Aldehyde dehydrogenase 1 (ALDH1), a marker of cancer stem cells, is also associated with metastasis and poor prognosis in many tumors. However, the prognostic value of either MACC1 or ALDH1 in non-small cell lung cancer (NSCLC) is unclear. In this study, we explored the relationship between MACC1 and ALDH1 expression, as well as their respective associations with clinicopathological features, to determine if either could be useful for improvement of survival prognosis in NSCLC.

**Methods:**

The expression levels of both MACC1 and ALDH1 in 240 whole tissue sections of NSCLC were examined by immunohistochemistry. Clinical data were also collected.

**Results:**

MACC1 and ALDH1 were significantly overexpressed in NSCLC tissues when compared to levels in normal lung tissues. Investigation of associations between MACC1 or ALDH1 protein levels with clinicopathological parameters of NSCLC revealed correlations between the expression of each with tumor grade, lymph node metastasis, and tumor node metastasis. The overall survival of patients with MACC1- or ALDH1-positive NSCLC tumors was significantly lower than that of those who were negative. Importantly, multivariate analysis suggested that positive expression of either MACC1 or ALDH1, as well as TNM stage, could be independent prognostic factors for overall survival in patients with NSCLC.

**Conclusions:**

MACC1 and ALDH1 may represent promising metastatic and prognostic biomarkers, as well as potential therapeutic targets, for NSCLC.

## Background

New lung cancer cases were estimated at 1.8 million and accounted for nearly 13 % of all new cancer cases in 2012, making it the most commonly diagnosed cancer worldwide [1]. It was also the most frequent cause of cancer-related death. Non-small cell lung cancer (NSCLC) accounts for approximately 85 % of all diagnosed lung cancers [2]; it has an overall 5-year survival rate of less than 20 % [2]. In China, the majority of patients diagnosed with NSCLC have advanced stage disease and are unsuitable for curative surgery.

The leading causes of cancer treatment failure are recurrence and metastasis. One gene that contributes to these processes is metastasis-associated in colon cancer-1 (MACC1). MACC1 is a critical regulator of the HGF/MET signaling pathway. It was first identified in colon cancer where it bound to the promoter of the MET gene to control its transcriptional activity [3, 4]. It has been shown to promote tumor cell migration and invasion in vitro and to induce tumor growth and metastasis in vivo [3, 5, 6]. MACC1 is considered an independent factor for prognosis and metastasis in colorectal cancer [3, 7]. Accumulating studies suggest that it could also be a prognostic and metastatic factor for other cancers, such as breast cancer [8], gastric carcinoma [9], hepatocellular carcinoma [10], renal pelvis carcinoma [11], malignant glioma [12], cervical carcinoma [13], and lung cancer [5].

Cancer stem cells (CSCs), also known as tumor-initiating cells, are a small population of cells within a tumor that have the capacity to self-renew and give rise to differentiated cell populations [14]. They are relatively resistant to chemotherapy and radiotherapy. These properties allow CSCs to repopulate tumors following treatment and lead to recurrence or metastasis [15–17]. Aldehyde dehydrogenases (ALDH) represent a family of enzymes located in the nucleus, cytoplasm, and mitochondria. ALDHs not only detoxify intracellular aldehydes or some cytotoxic drugs, but are also a key feature of CSCs [17–19]. ALDH1, which mainly promotes the conversion of retinaldehyde to retinoic acid, plays an important role in cell proliferation and differentiation in vitamin A metabolism [20–22]. Its overexpression can increase the risk of alcohol-related cancers [23]. Moreover, ALDH1 has been associated with metastasis and poor prognosis in many human cancers, such as breast cancer [24], ovarian cancer [17], lung cancer [18], and pancreatic cancer [25].

The involvement of MACC1 and ALDH1 in the recurrence and metastasis of NSCLC suggest that they could be valuable biomarkers for measuring disease progression and developing more accurate therapeutic strategies. To our knowledge, an association between MACC1 and ALDH1 in NSCLC has not yet been reported. In this study, we investigated the relationship between MACC1 and ALDH1 expression in patient tumor sections as well as compared their expression with the clinicopathology and prognosis of NSCLC.

## Methods

### Biopsy specimens

NSCLC tissues and adjacent noncancerous lung tissues were collected at the Department of Pathology of the First Affiliated Hospital of Bengbu Medical College, from January 2008 to December 2009. Patients who had received preoperative chemotherapy or radiotherapy, or other anti-cancer therapies, were excluded. All tissue samples were obtained with patient consent and the study was approved by the ethical committee of the Bengbu Medical College. The study group consisted of 240 patients, 160 males and 80 females, aged from 28–81 years; the average age was 58.3 ± 10.7 years. Tumor stage was assessed according to the 7th edition of the American Joint Committee on Cancer. Of the 240 NSCLC tissue samples, 33 were grade I, 157 were grade II, and 50 were grade III. As for histological type, 160 were characterized as squamous cell carcinoma while the remaining 80 were adenocarcinoma.

### Immunohistochemistry

Immunohistochemistry was performed according to the Elivision Plus detection kit instructions (Lab Vision, USA). Briefly, NSCLC- and corresponding normal lung tissues were fixed in 10 % buffered formalin and embedded in paraffin. Continuous 4 μm thick tissue sections were cut. All sections were deparaffinized and dehydrated with xylene and graded ethanol, then washed for 10 min in PBS (pH 7.2). Endogenous peroxidase activity was quenched by incubation of sections in methanol containing 3 % hydrogen peroxide for 10 min at RT, they were then placed in citrate buffer (pH 6.0) for antigen repair. After several washes in PBS, the sections were blocked with goat serum for 20 min at RT then incubated with mouse monoclonal antibody against human ALDH1 (Abcam, Cambridge, MA, USA) or rabbit polyclonal antibody against human MACC1 (Santa Cruz Biotechnology, Santa Cruz, CA, USA) for 1 h at 37 °C. All slides were counterstained with hematoxylin, dehydrated, air-dried, and mounted. Negative controls were prepared by omitting primary antibodies from the staining procedure. MACC1 and ALDH1 positive staining was mainly located in the cytoplasm of cancer cells.

### Evaluation of staining

Staining results were interpreted by two independent pathologists who were blind to clinical data and judged by semi-quantitative points. To overcome the intratumoral heterogeneity of antigen expression, ten visual fields from different areas of each NSCLC tumor were examined. If there was a disagreement, the observers would reexamine the section and reach a consensus [15, 26–28]. Staining was scored according to intensity and extent. The staining intensity score was graded as: 0, none; 1, weak; 2, moderate; and 3, strong. The extent of positive staining was graded as: 1, <10 %; 2, 11–50 %; 3, 51–75 %; and 4, >75 %. The intensity and extent scores were then multiplied to yield a final score that ranged from 0–12. Expression was considered positive when the score was ≥3. For tissues that were positive for both MACC1 and ALDH1, an average of the final score of each was taken.

### Statistical analysis

Relationships between either MACC1- or ALDH1 protein expression and clinicopathological variables were compared using Fisher’s exact test or Chi-square test. The association between MACC1 and ALDH1 protein expression was compared using Spearman’s coefficient test. The effects of MACC1 and ALDH1 expression on survival were determined by univariate and multivariate analyses. Independent prognostic factors were determined using the Cox regression model for multivariate analysis. The Kaplan-Meier method with log-rank test for univariate overall survival analysis was used to assess the relationship between the positive expression of either MACC1 or ALDH1 and clinicopathological factors using SPSS 19.0 software for Windows (Chicago, IL). A value of *P* < 0.05 was defined as statistically significant.

## Results

### Expression of MACC1 and ALDH1 in NSCLC, and their relationship to clinicopathology

**Fig. 1:**
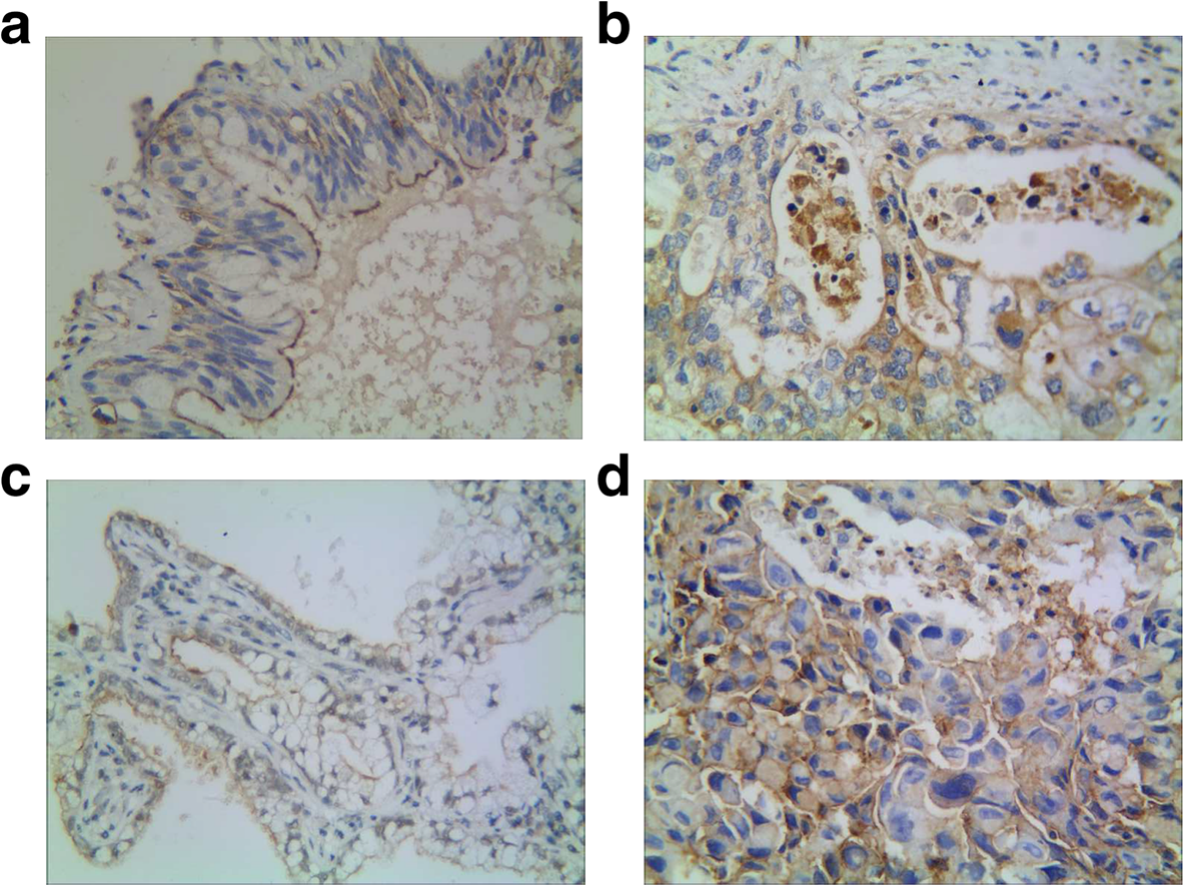
Representative results of MACC1 and ALDH1 in non-small cell lung cancer and control group. **a**: Control bronchiolar epithelial cells expressed MACC1 in the cytoplasm. **b**: MACC1 predominantly localized in the cytoplasm in moderately grade of squamous cell carcinoma (MACC1 × 400). **c**: Control bronchiolar epithelial cells expressed ALDH1 in the cytoplasm. **d**: ALDH1 predominantly localized in the cytoplasm in moderately grade of squamous cell carcinoma (ALDH1 × 400)

To evaluate the contributions of MACC1 and ALDH1 to NSCLC, their expression levels were assessed in both NSCLC and normal lung tissue sections using immunohistochemistry. These data were then compared to clinicopathological parameters. The positive rate of MACC1 protein expression was 64.2 % (154/240) in NSCLC tissues and 9.6 % (23/240) in normal lung tissues (Fig. 1a–b) and this difference was found to be statistically significant (*P* < 0.001). There were also significant differences between the positive expression of MACC1 and tumor grade (*P* = 0.015), lymph node metastasis (LNM) (*P* < 0.001), and tumor node metastasis (TNM) (*P* = 0.001). In contrast, there were no correlations detected between MACC1 expression and patient age (*P* = 0.622), gender (*P* = 0.341), tumor diameter (*P* = 0.490), location (*P* = 0.575), or histological type (*P* = 0.505).

Similar to MACC1, the expression of ALDH1 was significantly greater in NSCLC- than in control tissues, with positive rates of 55.8 % (134/240) and 12.5 % (30/240), respectively (*P* < 0.001) (Fig. 1c–d). There were also positive correlations between high expression of ALDH1 in NSCLC and tumor grade, LNM, and TNM (all *P* < 0.001). Furthermore, patients with squamous cell carcinoma had a higher positive rate of ALDH1 expression than did those with adenocarcinoma (*P* = 0.035). There were no associations detected between ALDH1 expression and patient age (*P* = 0.918), gender (*P* = 0.854), tumor diameter (*P* = 0.596), or location (*P* = 0.677) (Table 1).

**Table 1:** Correlation between the expression of MACC1 and ALDH1 and clinicopathololgical characteristics in NSCLC

Variable	MACC1		*P* value	ALDH1		*P* value
	negative	positive		negative	positive	
Tissue			<0.001			<0.001
Normal	217	23		210	30	
NSCLC	86	154		106	134	
Gender			0.341			0.854
Male	54	106		70	90	
Female	32	48		36	44	
Age (years)			0.622			0.918
< 60	38	63		45	56	
≥ 60	48	91		61	78	
Gross type			0.575			0.677
Central	60	102		70	92	
Peripheral	26	52		36	42	
Histological type			0.505			0.035
Squamous cell carcinoma	55	105		63	97	
Adenocarcinoma	31	49		43	37	
Diameter of tumor			0.490			0.596
< 3.0 cm	17	25		17	25	
≥ 3.0 cm	69	129		89	109	
Grade of tumor			0.015			<0.001
Well	19	14		26	7	
Moderately	53	104		70	87	
Poorly	14	36		10	40	
Lymph node metastasis			<0.001			<0.001
No	56	60		84	32	
Yes	30	94		22	102	
TNM stage			0.001			<0.001
I	18	14		25	7	
II	38	50		60	28	
III	30	90		21	99	
ALDH1 expression			<0.001*			
Negative	59	47				
Positive	27	107				

*positive correlation

### Univariate and multivariate analysis

**Fig. 2:**
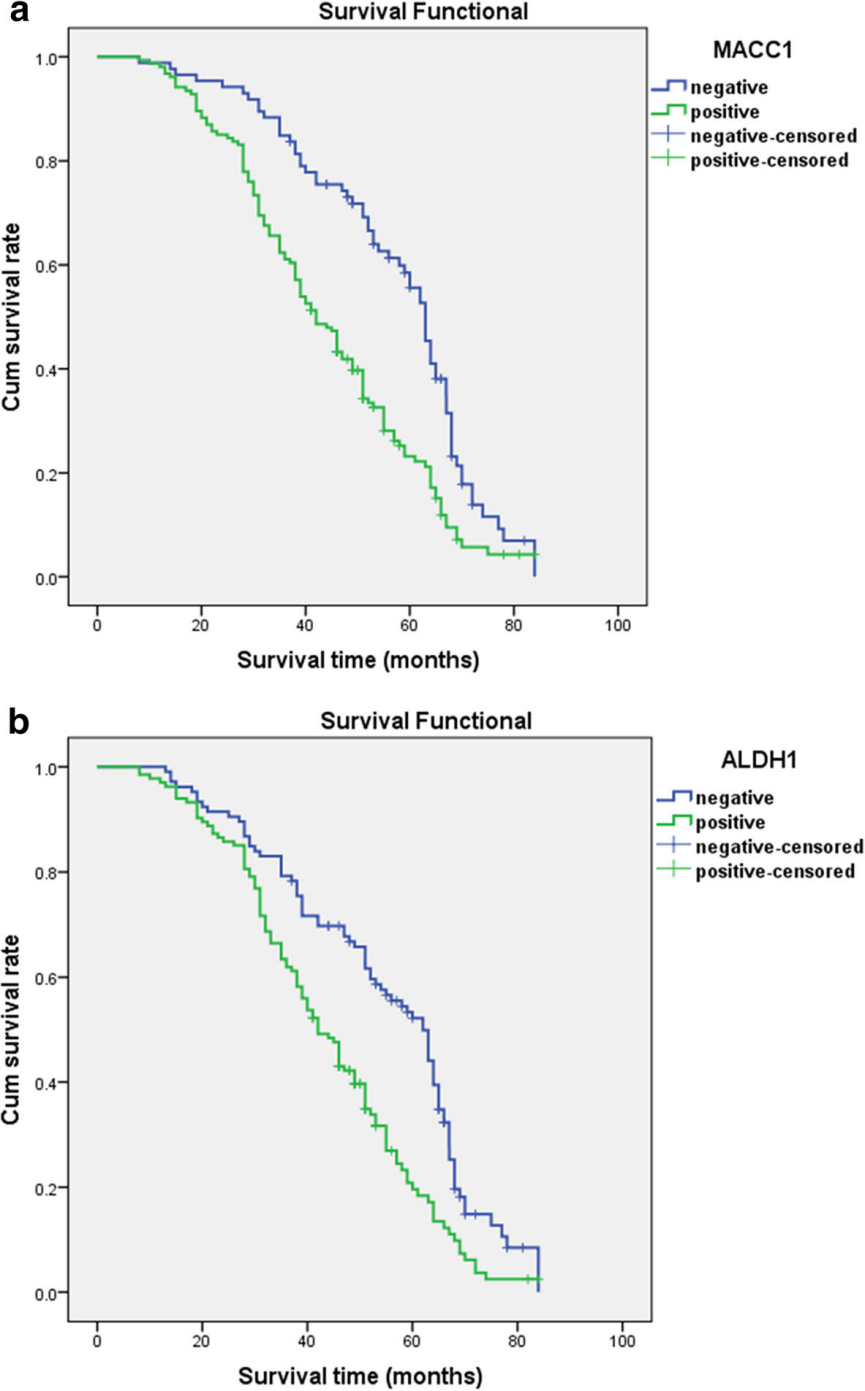
Kaplan-Meier survival analysis by MACC1 and ALDH1 status (*n* = 240). The y-axis represents the percentage of patient; the x-axis, their survival in months. The *green line* represents patients with positive expression of MACC1 (**a**) or ALDH1 (**b**) with a trend of worse survival time than the *blue line* representing the negative MACC1 group or ALDH1 group (*P* < 0.001). Mean survival time was 42.1 months for the positive expression of MACC1 group and 54.2 months for the negative MACC1 group. Mean survival time was 42.0 months for the positive expression of ALDH1 group and 52.1 months for the negative ALDH1 group

**Fig. 3:**
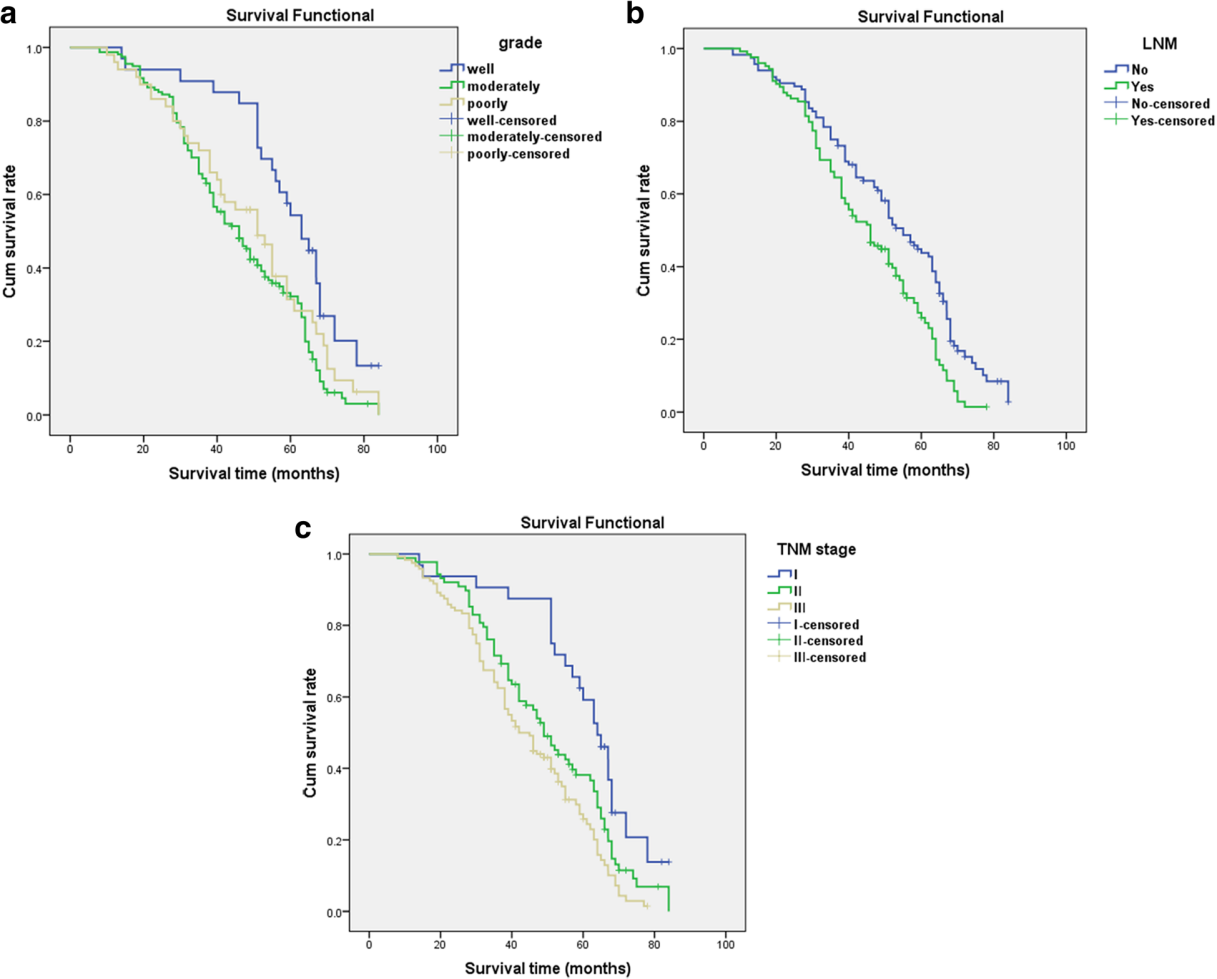
Kaplan-Meier survival analysis by grade, lymph node metastasis, and TNM stages status (*n* = 240). The y-axis represents the percentage of patient; the x-axis, their survival in months. **a** The *green line* represents patients with moderate grade of NSCLC with a trend of worse survival time than the *blue line* representing the well grade group (*P* < 0.001). Mean survival time was 44.1 months for the moderate grade of NSCLC group and 58.2 months for the well grade of NSCLC group. The *brown line* represents patients with poor grade of NSCLC with a trend of worse survival time than the *blue line* representing the well grade group (*P* = 0.027). **b** The *green line* represents patients with LNM of NSCLC with a trend of worse survival time than the *blue line* representing the no LNM group (*P* = 0.001). **c** The *green line* represents patients with stageII of NSCLC with a trend of worse survival time than the *blue line* representing the stageIgroup (*P* = 0.010). The *brown line* represents patients with stage III of NSCLC with a trend of worse survival time than the *blue line* representing the stage I group (*P* < 0.001). The *brown line* represents patients with stage III of NSCLC with a trend of worse survival time than the *blue line* representing the stage II group (*P* = 0.040)

Follow-up data showed that overall survival was significantly reduced in NSCLC patients with positive expression of MACC1 (42.1 months) compared to those who were MACC1-negative (54.2 months) (log-rank = 20.316, *P* < 0.001) (Fig. 2a). Similarly, the survival of ALDH1-positive patients (42.0 months) was significantly shorter than those whose tumors were negative (52.1 months) (log-rank = 17.065, *P* < 0.001) (Fig. 2b). Overall survival was also influenced by tumor grade, whereby NSCLC patients with low grade tumors survived significantly longer than those with tumors rated either moderate (log-rank = 12.826, *P* < 0.001) or poor (log-rank = 4.909, *P* = 0.027). There was no significant difference between the survival of patients with moderate or poor grade NSCLCs (log-rank = 1.524, *P* = 0.217) (Fig. 3a). Positive LNM was also linked with significantly reduced survival when compared with the no LNM group (log-rank = 11.148, *P* = 0.001) (Fig. 3b). The survival of stage I patients was significantly longer than that of those with either stage II (log-rank = 6.688, *P* = 0.010) or stage III NSCLC (log-rank = 16.359, *P* < 0.001). The survival of stage II patients was also significantly longer than that of those with stage III NSCLC (log-rank = 4.219, *P* = 0.040) (Fig. 3c).

Multivariate analysis showed that positive expression of either MACC1 or ALDH1, as well as TNM stage, were independent prognostic factors for NSCLC (Table 2).

**Table 2:** Multivariate survival analysis of 240 patients with NSCLC

Covariate	B	SE	*P* value	Exp (B)	95 % CI
MACC1	0.505	0.166	0.002	1.657	1.198–2.292
ALDH1	0.468	0.181	0.010	1.597	1.120–2.279
TNM stage	0.887	0.259	0.001	2.428	1.461–4.034

### Association between the expression of MACC1 and ALDH1 in NSCLC

Spearman association coefficient analysis revealed a positive association between the expression of MACC1 and that of ALDH1 (*r* = 0.368, *P* < 0.001).

## Discussion

NSCLC is a highly heterogeneous disease. This heterogeneity may affect the reproducibility of biomarker assessment [29, 30]. Thorough investigation of the prognostic value of candidate biomarkers is thus required to ensure validity. In this study, we analyzed MACC1 expression in NSCLC and matched normal tissues from 240 patients and compared it to clinicopathological parameters. We found that MACC1 expression was significantly greater in NSCLC tissues than in normal lung tissues. Moreover, it was positively associated with tumor grade, LNM, and TNM. Our findings are consistent with previous studies in NSCLC [31–35] suggesting that MACC1 could be useful as a clinical biomarker of NSCLC.

ALDH1, an intracellular enzyme related to retinoic acid, is widely regarded as a CSC marker in many cancers [18, 21, 25, 36–38]. In NSCLC, ALDH1 has been associated with carcinogenesis [39] and shown to predict a poor response to both chemotherapy and radiotherapy [28]. In our study, we found that ALDH1 expression was significantly related to tumor grade, LNM, and TNM. Furthermore, Kaplan-Meier survival analysis demonstrated that NSCLC patients with positive ALDH1 expression had significantly reduced survival compared with that of those negative for ALDH1. These findings suggest that ALDH1 plays an important role in the tumorigenesis, development, progression, metastasis, and prognosis of NSCLC. Several other immunohistochemical studies that examined the metastatic and prognostic significance of ALDH1 in NSCLC patients obtained similar results [18, 40, 41]. In addition, because squamous cell carcinomas tend to develop more rapidly than andenocarcinomas, could this suggest that ALDH1 is a good biomarker for more aggressive NSCLC. This is also consistent with a previous study [42]. Thus, our findings support the notion that ALDH1 would be a reliable biomarker of NSCLC, in particularly for predicting metastasis and disease progression.

Recurrence and metastasis are the most common causes of deaths in NSCLC. TNM staging is well-known as the guide for designing therapeutic strategies for patients with NSCLC, however, it provides limited information on the biological behavior of the disease. It is thus critical to find novel and effective prognostic strategies to predict recurrence and metastasis in NSCLC patients. In this study, multivariate Cox model analysis suggested that the positive expression of either MACC1 or ALDH1, as well as TNM stage, are independent prognostic factors for patients with NSCLC.

Abnormal ALDH1 expression may be involved in the initiation and recurrence of NSCLC through its involvement in CSCs. Among other things, self-renewal, proliferation, and an aptitude for multiple differentiation allow CSCs to induce angiogenesis and lymphangiogenesis to thereby access adequate nutrition and oxygen for rapid tumor growth. Indeed, the niche where CSCs reside mainly consists of vascular and lymphatic vessels.

Meanwhile, MACC1 contributes to tumorigenesis through the promotion of cancer cell proliferation and invasion through activation of the HGF/ Met signaling pathway [3, 4]. It is also involved in angiogenesis and lymphangiogenesis to promote tumor invasion and metastasis [43, 44]. In turn, these microvessels and microlymphantic vessels sustain CSCs that further promote tumor invasion and metastasis, thus creating a positive cycle of tumor advancement.

## Conclusions

Our findings suggest that aberrant expression of MACC1 and ALDH1 may play important roles in the development of NSCLC. The combined detection of MACC1 and ALDH1 may thus be valuable as markers for metastasis and thereby prognosis for patients with NSCLC.

## Abbreviation

ALDH1: Aldehyde dehydrogenase 1
CSCs: Caner stem cells
LNM: Lymph node metastasis
MACC1: Metastasis-associated in colon cancer 1
NSCLC: Non small cell lung cancer
PBS: Phosphate-buffered saline
TICs: Tumor initiating cells
TNM: Tumor-node-metastasis
